# Semi-Remote Gait Assistance Interface: A Joystick with Visual Feedback Capabilities for Therapists

**DOI:** 10.3390/s21103521

**Published:** 2021-05-19

**Authors:** Daniel E. Garcia A., Sergio D. Sierra M., Daniel Gomez-Vargas, Mario F. Jiménez, Marcela Múnera, Carlos A. Cifuentes

**Affiliations:** 1 Department of Biomedical Engineering, Colombian School of Engineering Julio Garavito, Bogota 111166, Colombia; daniel.garcia-a@mail.escuelaing.edu.co (D.E.G.A.); sergio.sierra@escuelaing.edu.co (S.D.S.M.); daniel.gomez-v@mail.escuelaing.edu.co (D.G.-V.); marcela.munera@escuelaing.edu.co (M.M.); 2 School of Engineering, Science and Technology, Universidad del Rosario, Bogotá 111711, Colombia

**Keywords:** human mobility, rehabilitation, smart walkers, joystick, visual feedback, teleoperation

## Abstract

The constant growth of pathologies affecting human mobility has led to developing of different assistive devices to provide physical and cognitive assistance. Smart walkers are a particular type of these devices since they integrate navigation systems, path-following algorithms, and user interaction modules to ensure natural and intuitive interaction. Although these functionalities are often implemented in rehabilitation scenarios, there is a need to actively involve the healthcare professionals in the interaction loop while guaranteeing safety for them and patients. This work presents the validation of two visual feedback strategies for the teleoperation of a simulated robotic walker during an assisted navigation task. For this purpose, a group of 14 clinicians from the rehabilitation area formed the validation group. A simple path-following task was proposed, and the feedback strategies were assessed through the kinematic estimation error (KTE) and a usability survey. A KTE of 0.28 m was obtained for the feedback strategy on the joystick. Additionally, significant differences were found through a Mann–Whitney–Wilcoxon test for the perception of behavior and confidence towards the joystick according to the modes of interaction (*p*-values of 0.04 and 0.01, respectively). The use of visual feedback with this tool contributes to research areas such as remote management of therapies and monitoring rehabilitation of people’s mobility.

## 1. Introduction

Physical rehabilitation (i.e., often referred to as physiotherapy) aims to restore people’s movement and physical functioning affected by injury, illness, disability, or traumatic events [1]. One of the main approaches for physical rehabilitation is targeted at the retraining of the human gait. Different health conditions can result in walking limitations or problems, such as accidents and neurological disorders (e.g., stroke, spinal cord injury, cerebral palsy), aging, musculoskeletal diseases (e.g., arthritis), heart disease, among others [2]. Depending on each patient’s condition, gait rehabilitation and assistance therapies might focus on providing, compensating, increasing, or retraining the lost locomotion capacities, as well as the cognitive abilities of the individual [2]. Specifically, training interventions seek to improve walking performance by (1) eliciting voluntary muscular activation in lower limbs, (2) increasing muscle strength and coordination, (3) recovering walking speed and endurance, and (4) maximizing lower limb range of motion [3]. In this manner, several techniques and approaches have been developed, ranging from overground and conventional gait training to robot-assisted and machine-based therapies [4,5].

In particular, robot-assisted gait training has gained considerable interest in recent decades since sensors and actuators allow safe, intensive, and task-specific therapies [6,7]. More profoundly, the integration of technology into physical rehabilitation processes also provide: (1) replicable therapies due to the recognition of movement patterns, (2) high intensity activities with adjustable difficulty, (3) monitoring of the patient’s physiological state, (4) virtual and augmented reality, as well as feedback strategies, can be used to provide immersive recovery experiences, (5) a trustworthy evaluation of the patient’s recovery success, and (6) reduction of the physical effort of the therapists [2,8,9]. Some examples of robotic solutions for gait rehabilitation are stationary or treadmill-based gait trainers, wearable devices (e.g., exoskeletons), ambulatory devices, and mobile robots (e.g., robotic walkers, standing devices) [7,10,11,12].

Robotic walkers are a potent rehabilitation tool; conventional walker’s have been empowered with features by integrating robotic and sensing technologies to allow the users to move and interact freely in a given environment [13]. As the conventional type, the robotic walkers improve overall balance, enhance lateral stability, and provide weight-bearing [14]. Moreover, robotic walkers are also capable of: (1) providing cognitive and physical support, (2) estimating biomechanical parameters, (3) extracting intentions of movement, (4) providing guidance and navigation, (5) generating feedback, and (6) allowing remote control [13,15,16]. Remarkably, this last feature is of great relevance since gait rehabilitation therapies often demand close accompanying of therapists to provide therapy monitoring [17].

Several studies have shown that combining robotic training with physical therapy might improve the recovery process of neurological patients [7,18,19,20]. It has been widely discussed that robots should not replace the neuro-rehabilitation therapy performed by a therapist [7]. In contrast, it has been established that rehabilitation robots such as robotic walkers can ease the physical burden on therapists. This has allowed the therapy manager to focus on more specific tasks such as functional rehabilitation and supervision of patients, optimizing their expertise and time [7,21,22]. In this sense, when using mobile robots such as walkers, it is crucial to provide the therapists with a communication channel that allows them to interact with the patient and the device without affecting independence or adding cognitive burden to the patient [17,23,24]. With such a communication channel, the therapists would command the robot remotely, perceive environment constraints, avoid hazardous situations, and monitor the patient state.

According to the above, this work reports the implementation and validation of two visual feedback strategies for the teleoperation of a simulated robotic walker, using a joystick during a path-following task. The first strategy allowed the users to visualize the path’s information on a computer screen and the obstacles to avoid. The second strategy used LEDs on the joystick to indicate if the user is in the appropriate direction. Thus, this work sought to determine which strategy was better in terms of path following error and usability. Additionally, this study also sought to identify the relevance for a group of healthcare professionals to be actively included during walker-assisted rehabilitation processes.

## 2. Related Work

This section presents the different applications of a set of technologies for the operation, control, real-time monitoring, and reprogramming of multiple devices. Typically, these devices are robots that enable and facilitate shared control tasks [25] in a fast, efficient, and safe way.

One of the main applications of teleoperation devices is drone control [26]. Due to their versatility, teleoperation devices can contribute to both military and healthcare [25,27], including environmental [28], and as a real-time monitoring mechanism [29]. Another interesting application of teleoperation is surgical systems, making possible minimally invasive human telesurgery over long distances [30,31]. Additionally, it should be noted that this type of device is essential in the control of semi-autonomous robots [32,33]. At this point, the implementation of interfaces that involved force or haptic feedback [34] for obstacle avoidance tasks in dynamic environments and assisted navigation [35,36] should be highlighted. Unfortunately, despite their adequate performance, this kind of application has shown calibration problems due to interface vibration [37].

After having a general overview of teleoperation devices’ applications, it is worth highlighting their incidence and impact on individual vehicles that favor the transportation of people who have permanently, totally, or partially lost motor skills, i.e., electric wheelchairs [38]. Due to its significant impact as an assistive device, interdisciplinary groups have been working on the development of novel interfaces to make electric wheelchairs more and more inclusive [39,40] as many people who suffer from tremors or spasms or are unable to control their movements completely, find it challenging to control wheelchairs with traditional joysticks. In this area, a particular and very significant application is joystick car driving for people with disabilities. Such a joystick driving device enables a person to drive a car while sitting in an electric wheelchair. The joystick’s action in the back and power direction govern a car’s acceleration or deceleration, while a steering wheel turns in the left and right direction [41]. Moreover, case studies about teleoperation devices in simulated environments have been reported in the literature, in order to mitigate as many errors as possible for the control devices when implemented in real-life cases [42]. Manipulators that recognize the intention of the user’s movement are also presented to make controlling the wheelchair easier [43]. Even control devices that implement haptic [44] and visual [45] feedback are given. Unfortunately, this technology is not designed to rehabilitate this kind of population but is limited to assisting. Considering the high impact of teleoperation devices, the need to include this kind of technology in rehabilitation and physical assistance scenarios should be highlighted.

This fact is also supported by the increasing demand for assistive robots, which require creating novel control modalities and interfaces to improve human–robot interfaces (HRI) [46]. These situations are generally characterized by collaborative work between robots and humans, where safe and efficient physical and cognitive encounters occur [47]. In particular, where humans and robots interact in complex scenarios where high performance is required [48,49], several strategies have been introduced, such as virtual environments [48], teleoperation with joysticks [50], interfaces with virtual impedance [50], and approaches to force feedback [51]. Thus, these kinds of methods have, for example, been used to interpret navigation commands and monitor robotic systems such as wheelchairs, exoskeletons, and mobile robots [52,53,54] cooperatively.

Some are presented to contextualize these proposed solutions with the strategies commonly used in smart walkers (SWs). To successfully and accurately facilitate the user’s knowledge flow, SWs incorporate various contact channels [15]. The key objective of these channels is to gather user-related information such as velocity, acceleration, location, force, torque, movement intention, among others [6]. SWs are fitted with interfaces that enforce control strategies to maximize their productivity to the fullest and, learn to respond effectively to the user’s stimuli [15].

In addition, SWs also provide guidance and aided navigation functions [55,56,57,58]. These characteristics include stability when leading the user through diverse and complex environments [6]. Some approaches are based on the path followed by methods where the ideal path is created offline, and SW is followed [59,60]. More dynamic methods, on the other hand, have also been applied, where path planning algorithms are used to estimate the desired path online (i.e., changing barriers and complex landscapes directly impact the intended path) [6,55].

The HRI paradigm has been independently discussed by recent implementations of SWs, such that SWs can communicate with the user and the environment safely and naturally. Similarly, using feedback modules to engage the individual in instruction activities directly, certain methods have mutual management strategies [55]. However, the qualitative evaluation of engagement techniques that have regular and intuitive mutual influence along the road to tasks are still lacking. In addition, visual interface cooperation was not completely used and exploited for guidance purposes in SWs, according to literature evidence. In this sense, this work describes the implementation and evaluation of two visual strategies on a joystick to guide with an SW.

The remainder of this work is organized as follows. Section 3 describes the robotic platform, the teleoperation device used during the study, and the proposed visual strategies. Section 3.3 presents the experimental setup, including the volunteers and trial description. Section 4 details the obtained results, presenting a comprehensive analysis of this work’s primary outcomes. Finally, Section 5 points out the concluding remarks and future works.

## 3. Materials and Methods

This section describes the proposed system for the robot teleoperation in terms of the included interaction platforms and the implemented feedback strategies. Likewise, this part also details the experimental protocol for the system’s validation, including quantitative and qualitative assessments.

### 3.1. Interaction Platforms

**Figure 1:**
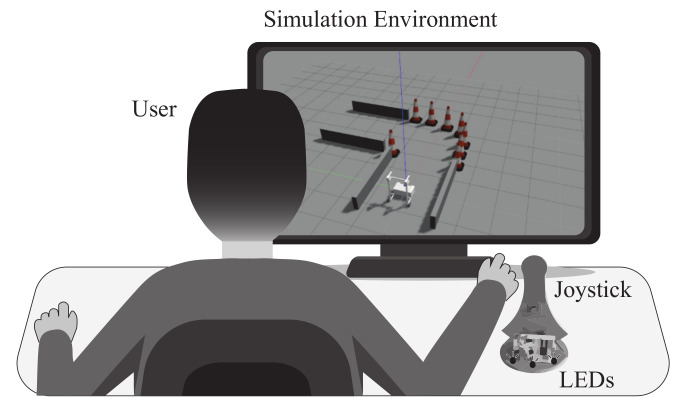
System proposed to provide visual feedback in teleoperation applications of smart walkers.

To provide visual feedback, the proposed system (see Figure 1) includes a (1) standard workstation to execute and control the simulation, (2) a joystick to provide teleoperation and feedback, and (3) a simulation environment to establish visual communication with the user.

#### 3.1.1. Standard Workstation

The system’s central compute consists of an Omen Laptop (HP, Palo Alto, CA, USA) integrated with an Intel Core i7-7700HQ of 8 cores (2.80 GHz) and a RAM of 16 GB. The device runs the Robotic Operating System (ROS, Kinetic Version) under a Linux distribution (Ubuntu 16.04-Xenial).

#### 3.1.2. Joystick

A Hapkit joystick (Stanford University, Stanford, CA, USA) was used, which provides a remote command interface. The Hapkit is an open-hardware joystick with one degree of freedom. The device was modified to include three LEDs placed on the base. These LEDs were added to provide a visual feedback strategy focused on showing how the user controlled the virtual smart walker (i.e., whether the robot’s trajectory was inside or outside of the proposed path).

#### 3.1.3. Simulation Environment

The graphic interface used the 3D visualization tool provided by the *Gazebo ROS* package (Gazebo ROS package. Link: http://wiki.ros.org/gazebo_ros_pkgs. Accessed on: 25 April 2021) and a 2D visualization tool, employing the *rviz ROS* package (Rviz ROS package. Link: http://wiki.ros.org/rviz. Accessed on: 25 April 2021). This way, the computer screen displayed the desired trajectory and the smart walker controlled by the joystick in real-time (see Figure 1). To simulate the smart walker motion, the Gazebo plugins for differential robots were used, and the Unified Robot Description Format (URDF) (URDF model. Link: http://wiki.ros.org/urdf/XML/model. Accessed on: 25 April 2021) was used to define the robot’s kinematics. The simulation measured the robot’s odometry and received speed commands through a speed controller provided by Gazebo. Moreover, a simulated laser rangefinder was also added to the robot, to provide obstacles sensing. The Gazebo plugin for laser rangefinder was also used. It is essential to highlight that for the joystick, the simulation, the admittance controller, the calculation of the kinematic estimation error (KTE), a sampling rate of 30 Hz was implemented.

#### 3.1.4. System Operation

**Figure 2:**
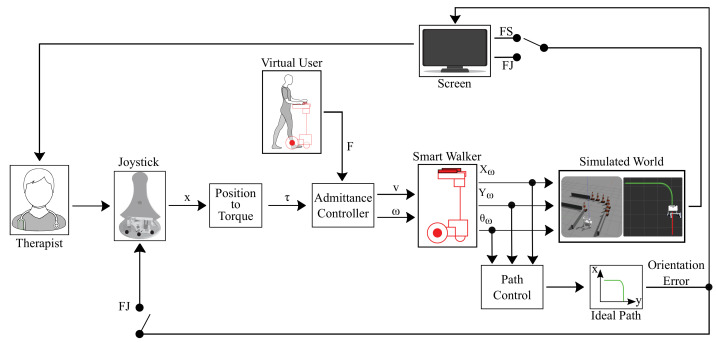
Illustration of the interaction system constituted by the feedback strategies, the path following task, and the simulation environment. *x* is the joystick position, τ is the virtual torque, F is the impulse force, *v* is the linear velocity, ω is the angular velocity, $X_{\omega }$ is the *x* coordinate of the walker’s position, $Y_{\omega }$ is the *y* coordinate of the walker’s position, and $\theta_{\omega }$ is the walker’s orientation. FJ refers to feedback on the joystick and FS to feedback on screen.

The therapists were asked to guide a simulated smart walker through a predefined environment (see Figure 1). Initially, the system indicated to the participants how to control the smart walker by showing them the simulated environment (see Figure 2). The robot was rendered in such a way that it resembled the standard structure of a robotic walker. To simulate the patient, a constant impulse force (F) on the robot was generated. For this specific case, this force was decided as a constant parameter. The task of the therapists was to control the turning of the robotic walker. To this end, virtual torques were generated by moving the Hapkit from one side to the other, as Figure 2 shows. Specifically, the position of the joystick was converted to torque through the implementation of Equation (1):

(1) $$\tau =\left(-1\right)*k_{1}*tanh\left(\frac{x}{k_{2}}\right)$$

where τ is the torque, $k_{1}$ a gain with a value of 5000, $k_{2}$ a gain with a value of 50, and *x* the joystick position. This formulation was based on a previous work for guiding people with virtual torque signals, using a smart walker [61]. Subsequently, a constant virtual force (F) of 10 *N* was generated to simulate a user driving the robotic walker. In this way, the force (F) and torque (τ) were used to generate linear (v) and angular (ω) velocities using an admittance controller [6,59].

Finally, two feedback modes on the joystick were tested during the simulation: (1) feedback on the screen and (2) feedback on the joystick (i.e., FS and FJ, respectively). It should be noted that through the positions in *x* (Xω) and *y* (Yω) and the virtual robot’s orientation (θω), the path’s orientation error, achieved by the device concerning the proposed trajectory, can be estimated.

In addition to the obstacles placed in the simulation environment, the robot proposed an ideal path to be followed by the robot. Thus, the odometry of the robotic walker was used to estimate the path-following error. To obtain the correct direction of turning at each pose of the path, the path following controller developed by Andaluz et al. was used [62].

### 3.2. Visual Feedback Strategies for Teleoperation

**Figure 3:**
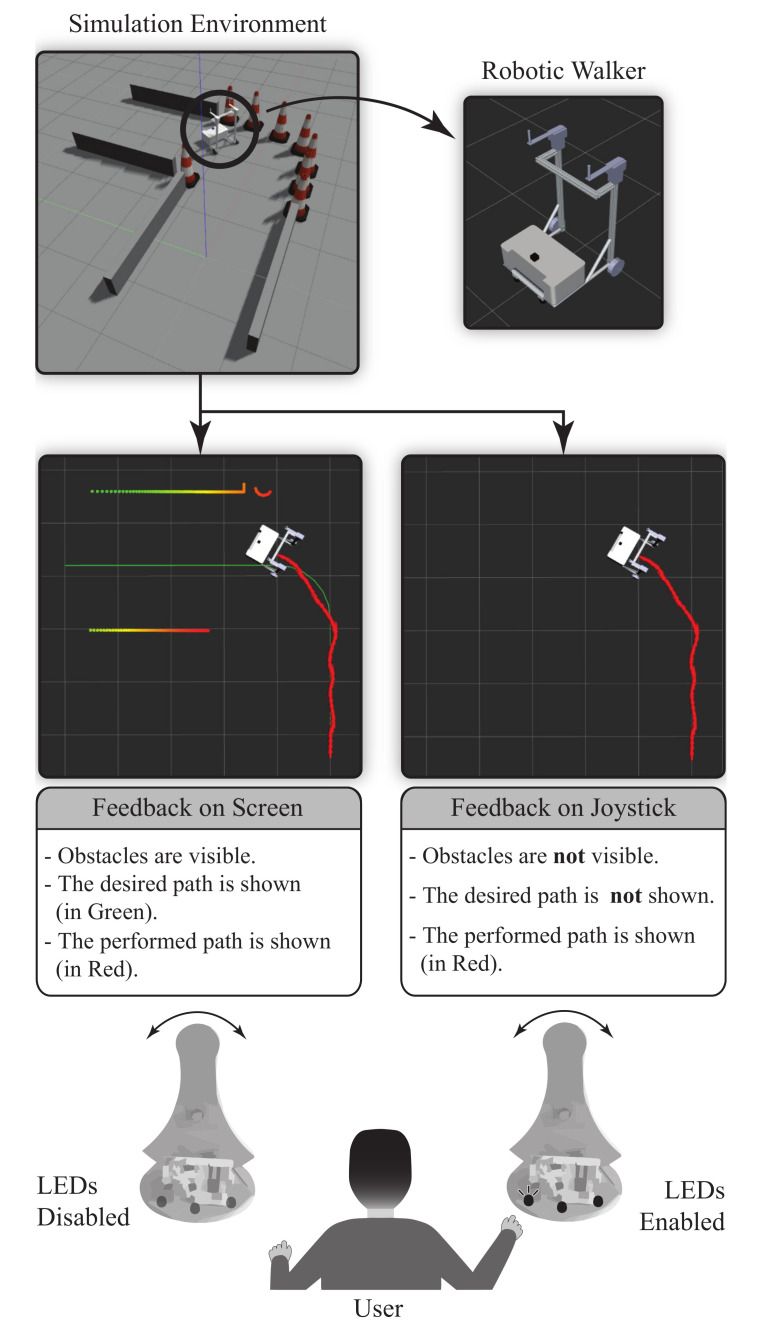
Visual feedback strategies applied in the robotic walker teleoperation. The upper figures show the simulation environment (gazebo) and the robotic walker used in the system. The central figures illustrate the ideal path and the proposed strategies with their characteristics in the graphic interface. The lower part exhibits the action on the joystick for each method.

Figure 3 shows the proposed visual feedback strategies for the robotic walker’s teleoperation in the simulation environment from the interaction platforms detailed in the previous section. Specifically, the strategies comprise (1) feedback on the screen and (2) feedback on the joystick.

#### 3.2.1. Feedback on the Screen (FS)

For this modality, the user receives the feedback directly from the graphic interface. Therefore, the ideal and current paths are exhibited on the screen so that the therapist can correct the smart walker’s trajectory by moving the Hapkit. The virtual walker and the performed path were updated every 50 ms, approximately. Moreover, the obstacles sensed by the laser rangefinder are also displayed.

#### 3.2.2. Feedback on the Joystick (FJ)

**Figure 4:**
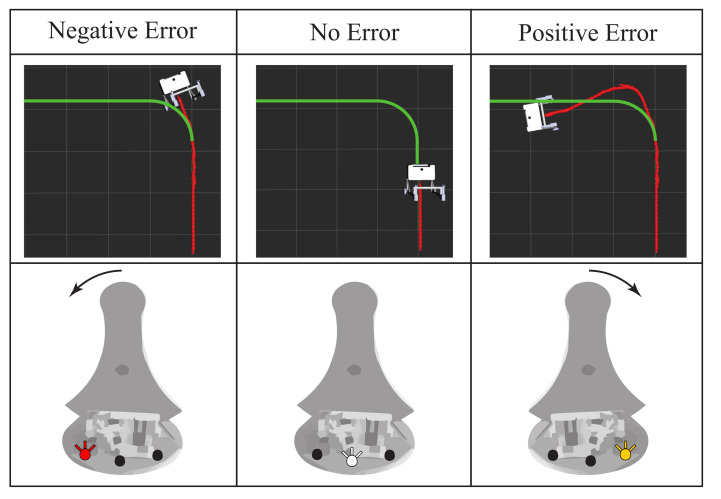
Illustration of the feedback strategy on the joystick. Three LEDs placed on the base of the device indicate the existence or absence of a path following error. The arrows indicate how to move the joystick to correct the error. The desired path is shown in green. The achieved path is shown in red.

In this mode, three LEDs located on the base of the Hapkit provide information about the path-following error. Specifically, neither the obstacles nor the desired path are displayed on the graphic interface. A red LED placed on the left side indicates negative errors concerning the ideal path, a white LED in the middle illustrates when the smart walker is correctly oriented, and a yellow LED placed on the right side indicates positive errors (see Figure 4). In this way, this strategy’s primary goal consists of keeping the white LED (i.e., placed in the middle) switched on as long as possible.

More precisely, as can be seen in Figure 4, a negative error experienced in the virtual walker implied a deviation to the right side concerning the ideal trajectory. In this case, the joystick turned on the left LED (red light), indicating to the users that they should move the control in that direction to keep the robot inside the trajectory. Similarly, this process occurred for the positive error when the walker was in the left part of the proposed path (see Figure 4). Thus, the joystick turned on the yellow light (right LED), indicating that the user should correct the walker’s trajectory. Finally, the center LED (white light) was turned on for a no-error state, showing that the user controlled the robot correctly, as Figure 4 shows.

In addition, for the LEDs to light up, at least three successive data samples had to have the same error behavior, i.e., for the yellow LED to light up, at least three consecutive data samples had to have a positive error. Finally, it is worth noting that for this mode, a threshold was defined for making the task a little more user-friendly and thus, to be able to correctly determine the error on the trajectory. This threshold was 10 degrees both to the left and to the right. That is when the subject deviated from the proposed route and exceeded this threshold, the respective LEDs would light up. The threshold was defined experimentally to avoid overloading the cognitive communication channel between the device and the user.

### 3.3. Experimental Protocol

This section describes the experimental validation executed to evaluate the performance of the feedback strategies presented above.

#### 3.3.1. Participant Recruitment

Considering the goal of the system, 14 occupational therapists participated in this study. The group was composed of 12 females and 2 males with an average age of 23.4±1.8 y.o. and a mean clinical experience of 2.3±1.2 years. Table 1 summarizes the demographic information of the participants recruited according to the exclusion and inclusion criteria shown below:

- Inclusion Criteria: Occupational therapists (OT) or last year students in occupational therapy (OT Student) with experience in gait rehabilitation scenarios.
- Exclusion Criteria: Candidates who presented upper-limb injuries, cognitive impairments, or any condition that impedes using of the joystick and the graphic interface were excluded in this study.

#### 3.3.2. Experimental Procedure

Before the experiment, participants were asked to fill out a brief three-question questionnaire (i.e., Have you worked with walkers? Have you worked with robotic walkers? Have you worked with assistive robotics, in general?) to determine the level of approach they have had with this type of devices. This questionnaire had two answer options, yes if they had some approach to this type of devices, and no in case they do not have any previous experience.

All participants were given appropriate instructions on the operation of the two feedback strategies prior to the trials execution. The order in which the feedback strategies were used, was randomized for each participant. Subsequently, the simulation environment was set up with a left-turn trajectory to analyze and compare the effects of the two methods. Each participant was required to complete three attempts of the path-following task, and only the third one was used for analysis purposes. The first and second attempts were used for training. A resting period of 30 s was allowed between each attempt of the same feedback mode, whereas a resting period of 1 min was allowed when the feedback mode was changed.

Moreover, a maximum execution time of 1 min and 30 s was allowed for each attempt. In case of exceeding this time, the attempt was aborted. The participants were only asked to attend one session.

During the trial, log files were stored, and the rosbag ROS package (Rosbag ROS package. Link: http://wiki.ros.org/rosbag. Accessed on: 25 April 2021) was used to record the robotic walker information and the movements of the joystick. Once they accomplished each strategy, the participants completed a qualitative survey to assess the acceptance and usability of the proposed system.

#### 3.3.3. Quantitative Assessment

To measure the users’ performance during the trials, the kinematic estimation error (KTE) was calculated in this study [63]. The KTE compares the achieved path by the participant against the ideal (the proposed path for the experiment), calculating the mean error and including the trial variance, as Equation (2) shows.

(2) $$KTE=\sqrt[{}]{|\bar{\varepsilon }{|}^{2}+\sigma^{2}},$$

where the $|\bar{\varepsilon }{|}^{2}$ value refers to the mean squared errors between the ideal and achieved paths, and the $\sigma^{2}$ value represents the data variance [24,63]. It is worth noting that this equation does not require the walker’s speed or acceleration, since it aims to provide insights into the spatial performance of the path-following error, rather than kinematic information. Furthermore, the virtual impulse force was simulated as constant, thus the linear velocity, generated by the admittance controller, was also constant. For this reason, the KTE is used to estimate the error between the proposed trajectory and the one achieved by the subject.

Moreover, to analyze the user–joystick physical interaction during the task, several kinematic characteristics such as the duration [s], the distance [m], the orientation error [rad], the correction torque [N.m], and the walker’s pose (i.e., $X_{\omega },Y_{\omega },\theta_{\omega }$) were recorded. Notably, the indicator related to the correction torque indicates the therapist’s average torque, when moving the Hapkit. These indicators were only estimated for the third trial of each mode, i.e., the validation trial.

#### 3.3.4. Qualitative Assessment

Based on previous studies related to qualitative assessments in applications using smart walkers, as presented in [6,59,64], this study included a perception and usability survey. Table 2 illustrates the questionnaire adapted to this study to assess the user interaction with the system. The questions were intended to estimate the naturalness, intuition, and user preference concerning the proposed strategies. For that, the questionnaire integrated six categories: Facilitating Conditions (FC), Performance and Attitude Expectation (PAE), Expectation of Effort and Anxiety (EA), Behavior Perception (BP), Trust (TR), and Attitude towards Technology (AT). Moreover, the survey integrated a 5-point Likert scale to score the questions, being five fully agreeing and one completely disagreeing. As described in Table 2, some questions were negatively formulated. Regarding these questions, the collected answers were mirrored along with the neutral scale value (i.e., score = 3) for analysis purposes.

To analyze the results of this survey, it was necessary to compile each category’s questions into a single number. To achieve this, the percentage of each point of the Likert scale was calculated concerning the total number of responses for each mode. That is, for the specific case of FC, we calculated the quotient between the sum of the number of votes for totally disagree (for the 4 questions) and the number of possible votes for the mode. This last value for this case is 56 since there are 14 participants and 4 questions. Finally, this quotient was multiplied by 100 to obtain its equivalent in percentage. This procedure was applied to both modes in each of the categories.

#### 3.3.5. Statistical Analysis

For the quantitative data, the Shapiro–Wilk test assessed the normality of the measured characteristics, and the t-student test determined whether there were significant differences between the proposed strategies. Likewise, in the quantitative assessment, the Mann–Whitney–Wilcoxon (MWW) test assessed statistical differences between the proposed feedback methods. Thus, for this case, the test was used because of data reported to have minimal Type I error rates and equivalent power without testing for Likert [65,66]. A significance value of *p* < 0.05 was used for all the statistical tests.

#### 3.3.6. Ethics Statement

The Research Ethics Committee of the University approved this experimental protocol. The participants were informed about the experiment’s scope and purpose, and their written informed consent was obtained before the study. The participants were free to leave the study when they decided to do so.

## 4. Results and Discussion

This section describes and discusses the primary outcomes of this study regarding quantitative and qualitative results. A total of 14 sessions were completed, and no collisions occurred during the simulations.

**Figure 5:**
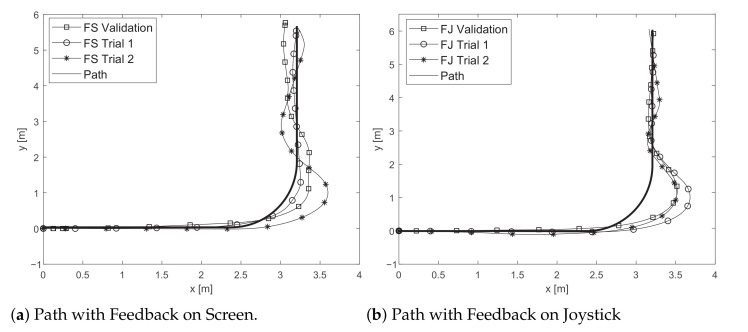
Path following task examples for one subject. Training and validation trials for (**a**) feedback on the screen and (**b**) feedback on the joystick are shown.

Figure 5 illustrates the results registered by a participant during the different trials with the two proposed feedback strategies. These results were selected, as the participant exhibited an average performance, in comparison to all participants. The upper figure shows the achieved trajectories using the feedback on the screen method, and the lower part displays the paths for the feedback on the joystick. Trials 1 and 2 refer to the trajectories obtained in the training stage, and the validation represents the path used to extract the kinematic and interaction data exhibited below.

It is worth noting that a single, simple path was proposed to validate this teleoperation tool. This fact is supported by the data provided by the literature on the cognitive load produced by visual interfaces when they are poorly implemented [67,68]. Several authors recommend that to validate this type of technology, simple tasks should be performed so that users become familiar with the work to be done [69,70] and thus, gradually increase the complexity of the task. For this reason, since the joystick and the visual strategies are in a validation stage, such a route was designed to have a clear perception of the clinicians regarding the tool.

In addition, it should be noted that the experiment was conducted in a simulated environment. Considering what the literature suggests about mobile robots, simulations play an essential role in system validation, as presented in [71]. Some authors say that although it has been shown that it is possible to train the devices in real environments, the amount of trials needed to test the system discourages the use of physical robots during the training period [71,72]. Therefore, it is recommended to validate the robot performance in the early stages in simulated environments to mitigate as many errors as possible that may occur in the real application [72].

### 4.1. Quantitative Results

Table 3 summarizes the mean values of the characteristics obtained in this study to measure the participants’ performance during both strategies. The measured indicators comprise aspects such as the duration to accomplish the path, the distance traveled by the robotic walker, the kinematic estimation error (KTE), the orientation error, and the correction torque calculated from the joystick movements.

In the statistical context, the Shapiro–Wilk test determined that all parameters followed a normal distribution. Therefore, to find significant differences between the modes (i.e., FS: feedback on the screen and FJ: feedback on the joystick), the t-student test was performed. Notably, all measured parameters registered statistically significant differences (see Table 3). Hence, it can be stated that each feedback methodology provides an entirely different teleoperation performance. In this regard, although the path was the same for both strategies, the interaction parameters evidenced statistically significant changes.

In terms of duration and distance, the feedback on joystick (FJ) strategy showed a decrease in the mean value compared to the feedback on screen (FS) mode. Thus, it can be highlighted that the therapists performed better trajectories (i.e., closer to the reference path) when the joystick provided visual feedback. In addition, this behavior also led to the accomplishment of the path in shorter times. This result may be supported by the fact that the joystick’s feedback mode required fewer correction torques on the device (see Table 3). Moreover, the LEDs’ use as visual feedback provides an instantaneous indicator of the path following error, compared to the error’s perception on the screen.

Regarding the KTE error, the feedback mode on the joystick (FJ) registered the lowest values. This result suggests that the user–device interaction was more intuitive and efficient in keeping the walker within the proposed path, concerning the FJ strategy. In addition, the comparison between the strategies was evidenced by statistically significant differences, which was expected, considering that the values obtained from the FS were always considerably higher.

Similarly, the orientation error presented lower values for the FJ strategy. Thus, this result indicates that the volunteers managed to keep the robotic walker within the ideal path more easily. In contrast and similarly to the previous results, the feedback on the screen presented higher error values.

Finally, regarding the correction torque, the FS strategy exhibited the highest values. This result could be supported by the fact that this mode demanded more correction movements with the Hapkit. In contrast, when the therapists controlled the smart walker using the FJ strategy, the parameters evidenced lower values, indicating that the joystick’s feedback was more efficient. In statistical terms, significant differences were found between the modes.

Comparing these results with literature, in [73] negative results were obtained when the user received feedback on a screen in a path-following task. Moreover, the study by [24] suggested that visual feedback on the joystick was better, even compared with haptic feedback. This evidence suggests that visual methods can be implemented to facilitate the therapists’ involvement and to provide a useful teleoperation tool. Furthermore, ref. [74] emphasized the importance of including an efficient visual strategy for teleoperation applications, the proposed system’s results suggest that the feedback on the joystick could be a solution with potential use in this area.

### 4.2. Qualitative Results

This study included a preliminary survey to assess levels of knowledge and perception of robotic technology application in rehabilitation settings. Overall, 58.3% of the participants had worked at least once with conventional walkers. However, 91.7% of the therapists had never interacted with robotic walkers, and 66.7% said they had not used any robotic devices for assistive applications. These results support the need to actively, closely, and safely [20,75] have therapists during robotic walker therapies [23,76]. Furthermore, such inexperience on the part of the therapists may be related to the low development of tools to facilitate their task in the course of their therapy [50,77].

**Figure 6:**
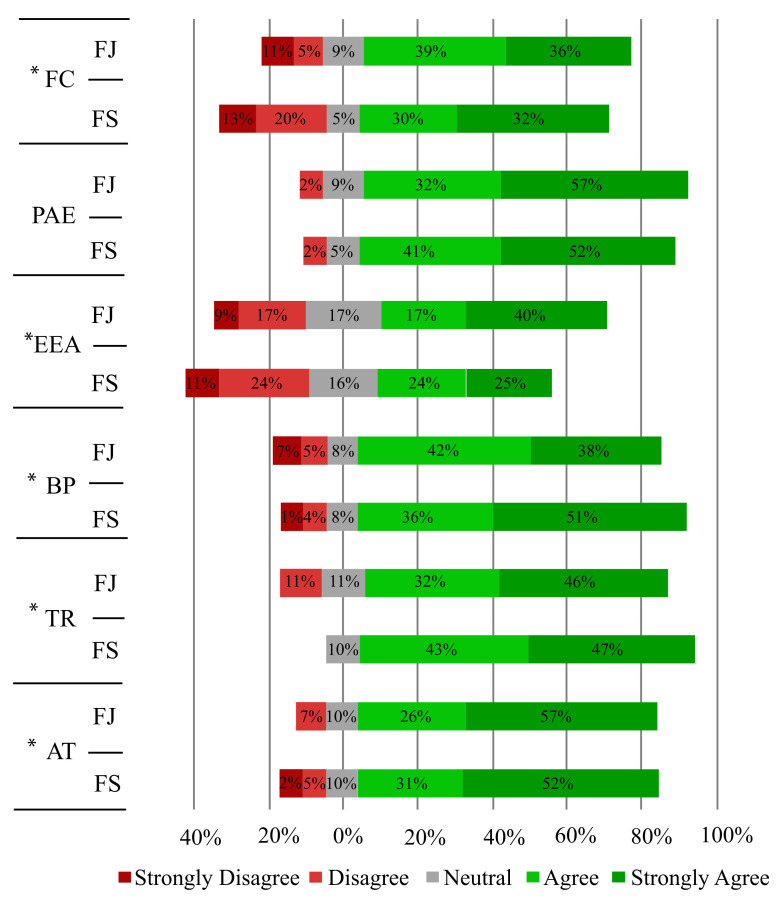
Distribution of acceptance and usability questionnaire answers. Feedback on screen (FS), feedback on joystick (FJ). Asterisks indicate that there are significant differences between modes.

On the other hand, to determine the naturalness, intuitiveness, safety, perception, complexity, and users’ preference with the proposed strategies, a questionnaire (see Table 2) was accomplished by all participants. Figure 6 summarizes the answers for the different categories of the implemented questionnaire.

In the statistical context, the Mann–Whitney–Wilcoxon (MWW) determined significant changes between both assessed feedback strategies. Table 4 summarizes the results for the MWW test applied between the interaction strategies and the questionnaire categories.

In particular, the questions in the category of facilitating conditions (FC), which assessed aspects such as safety, ease of use, and attitude during the interaction, show a mainly positive distribution. For the mode on the screen, the perception was slightly higher than the feedback strategy on the joystick (see Figure 6). Although, in general terms, this aspect was positive for both methods. Furthermore, volunteers indicated ease to interact with the proposed system independently of the applied modality. This way, the results confirm that the strategies implemented were adjusted, generating non-complex scenarios for the users.

Regarding the Performance and Attitude Expectancy (PAE) category, these questions were intended to assess the device’s overall performance. The distribution of responses for this category is positive and uniform (see Figure 6). This result indicates that users showed a favorable attitude and acceptance for both modes, which is confirmed by the no significant differences between the groups (i.e., FS and FJ) shown in Table 4.

Concerning the category of effort and the perception of anxiety (EEA), the statistical trial revealed significant differences between the feedback modes. Thus, the screen strategy presented better results in comparison with the feedback on the joystick. Moreover, although the tendency was positive for most participants, some therapists perceived considerable anxiety and relevant efforts using the system.

For the perception of behavior (i.e., a category that aimed to measure the user-device communication directly), there were significant differences between the proposed strategies, as Table 4 shows. Moreover, Figure 6 illustrates the distribution for both cases, where the feedback on the joystick exhibited more positive values than the method on the screen. This result indicates that volunteers felt more comfortable and confident using this strategy.

In the TR category case, which assessed the confidence of the subjects when using the device, strategies evidenced differences between them (see Figure 6). This result is consistent with the statistical analysis exhibited in Table 4. Specifically, the joystick’s feedback mode presented a more extensive positive distribution than the method on the screen. Thus, the favorable perception could be supported because the subjects felt more confident interaction under the guided feedback mode using LEDs, possibly because this strategy could be more natural and intuitive in teleoperation applications.

Regarding to the category focused on measuring of the subjects’ attitude towards technology (TA), there was a slight decrease in the interaction mode showing the orientation error on the screen (Figure 6). Table 4 shows statistical differences between the strategies, where the method on the screen registered lower favorable perception. Moreover, the positive distribution in the joystick’s feedback strategy indicates that users understood the device’s teleoperation satisfactorily employing LEDs for feedback information.

It is worth mentioning that one of the significant limitations of this study is related to the path chosen for the experimental trials. However, this work’s main objective was to validate the strategy in a simple scenario, while further works will include more complex experimental conditions. Exceptionally, it would be useful to include obstacles, longer and more difficult paths, and a real smart walker.

Furthermore, it is important to highlight a key point within this study related to the feedback strategies. If the joystick’s feedback lights were not on the device but on the screen, very similar results would probably be obtained. However, the idea of this study was to validate two methods of feedback and to verify whether the mode with less cognitive load would allow users to obtain better results in the path-following task. Additionally, in future implementations we expect to develop a portable joystick that can be carried by the therapist. In this way, the device will not be required to be connected to a desktop computer or workstation. Therefore, with this work we sought to validate a feedback method that applies to this portable version.

## 5. Conclusions and Future Work

A new method for walker-assisted gait therapy monitoring and control using a command interface was proposed in this article. Using the visual capability of a joystick device, a physical and cognitive communication channel was developed. In this sense, a Physical and Cognitive Interface (PCI) for human–robot interaction between therapy manager and joystick was created in this work. In addition, different levels of communication were provided by a series of visual feedback strategies.

On 14 participants who completed multiple trials with the device, an acceptance and usability questionnaire was applied. Participants had a higher level of confidence in the visual feedback mode using the joystick’s LEDs, as well as a greater understanding of the interaction. Similarly, the kinematic estimation error (KTE) was determined during experimental trials, with lower values in this strategy.

The use of feedback strategies integrating physical and cognitive interaction between the therapist and an interface contributes to research areas such as telerehabilitation and monitoring of people in hospital environments. Likewise, those applications empower therapist capabilities by reducing the energetic expenditures performing physical activities. Moreover, through the system’s information, the therapist can perceive the patients’ perceptions using mobile devices for assistive applications. This way, the therapist can control the SW and prevent undesirable situations such as falls or collisions.

As a result, on the one hand, the therapist would have a greater view of the environment and people’s recovery process with the proposed tool. Overall, there were some shortcomings due to participants who did not understand the joystick interface channel used for feedback. On the other hand, learning how to interpret therapy knowledge through a non-traditional communication medium can necessitate a brief period of training.

As future work, the implementation of the device in a real environment with slightly more complex path-following tasks will be carried out. For that reason, the idea of this study was also to develop an innovative tool in the context of teleoperation in robotic walkers. This tool was designed to explore an alternative to conventional remote control devices for walkers (e.g., laptops, tablets). In particular, they tend to have complex and unfriendly interfaces, thus generating considerable cognitive load for clinicians and not allowing them to adequately perform their role within the therapy.

## Figures and Tables

**Table 1:** Demographic information of the participants involved in the study.

Subject	Age (Years)	Gender	Occupation	Experience (Years)
1	21	Female	OT Student	1
2	23	Female	OT Student	1
3	23	Female	OT Student	2
4	21	Female	OT	2
5	23	Female	OT	3
6	22	Male	OT Student	1
7	21	Female	OT	3
8	27	Female	OT	5
9	23	Female	OT	3
10	24	Female	OT	4
11	24	Male	OT	2
12	25	Female	OT	2
13	25	Female	OT	3
14	25	Female	OT	1

**Table 2:** Perception and usability questionnaire implemented in the experiment. Asterisks indicate that questions were formulated negatively.

Cat.	No.	Question
FC	1	I had the necessary knowledge to use the device.
	2	I have previously used similar systems.
	3	The training was enough to understand the behavior of the mode.
	4	Before using the device, I was intimidated. *
PAE	1	If I had to use a joystick as a command interface, this device would be useful to me.
	2	If I had to use a joystick as a command interface, I would like to use this device.
	3	Using this device improves my ability to use command interfaces.
	4	Similar devices may allow a new form of therapist-patient interaction.
EEA	1	In this mode, learning to operate the device was easy.
	2	In this mode, I think I quickly learned to control the device.
	3	In this mode, I was afraid of making mistakes or breaking something. *
	4	If I had to control a robotic walker with this device in this mode, I would be afraid of losing control. *
	5	In this mode, working with the device was so complicated, which is hard to understand. *
BP	1	In this mode, I felt the device understood me.
	2	In this mode, I felt the device communicate with me.
	3	In this mode, I felt like I was controlling the virtual walker with the device.
	4	In this mode, I felt that the device helped me control the virtual walker.
	5	In this way, I believe the type of feedback was appropriate and effective.
	6	In this mode, I think the kind of feedback was easy to understand.
TR	1	In general, I would trust when the device gives me advice on how to control the virtual walker.
	2	In general, if the device give me advice, I would follow it.
AT	1	In this mode, I had fun using the device.
	2	In this mode, I think it is interesting how the device interacts with me.
	3	In this mode, using the device was frustrating for me. *

**Table 3:** Summary of kinematic and interaction data obtained during the trials. All parameters followed a normal distribution. Highlighted parameters (in gray) evidenced significant differences between both strategies (*p* < 0.05). Asterisks indicate that the data have a normal distribution.

Parameter	FS	FJ	*p*-Value
Duration [s]	26.15 ± 2.58 *	25.23 ± 3.91 *	<0.01
Distance [l	3.81 ± 2.20 *	3.80 ± 1.92 *	0.04
KTE [m]	0.31 ± 0.06 *	0.28 ± 0.03 *	0.02
Orientation Error [rad]	0.35 ± 0.11 *	0.32 ± 0.06 *	0.03
Correction Torque [N.m]	1.37 ± 3.28 *	1.26 ± 3.75 *	<0.01

**Table 4:** *p*-values obtained from the Mann–Whitney–Wilcoxon test. The highlighted values (in gray) indicate differences with a significance level of 0.05.

Category	Feedback on the Screen vs. Feedback on the Joystick
FC	0.01
PAE	0.50
EEA	0.03
BP	0.04
TR	0.01
AT	0.02
